# Factors Associated With Medication Compliance in Elderly Patients With Type 2 Diabetes Mellitus: A Cross-Sectional Study

**DOI:** 10.3389/fpubh.2021.771593

**Published:** 2022-01-11

**Authors:** Nobuyuki Wakui, Mizue Ozawa, Takahiro Yanagiya, Saki Endo, Chikako Togawa, Raini Matsuoka, Shunsuke Shirozu, Yoshiaki Machida, Mayumi Kikuchi

**Affiliations:** ^1^Division of Applied Pharmaceutical Education and Research, Faculty of Pharmaceutical Sciences, Hoshi University, Tokyo, Japan; ^2^Shinagawa Pharmaceutical Association, Tokyo, Japan

**Keywords:** elderly patients, type 2 diabetes, medication compliance, quality of life, knowledge of drug effects, knowledge of side effects, Cramer's V

## Abstract

The average age of patients with type 2 diabetes in Japan is over 70 years. Elderly patients tend to have poor medication compliance, therefore, it is important to understand their individual situations to improve medication compliance, the treatment of their diabetes, and their quality of life (QOL). This study aimed to identify factors associated with medication compliance in elderly type 2 diabetic patients. A cross-sectional study based on questionnaires was conducted on type 2 diabetes patients aged 65 years or older. The participants were recruited from patients who visited three dispensing pharmacies in the Shinagawa area of Tokyo between March 1 and September 30, 2019. The questionnaire consisted of patient information (sex, age, medication compliance status, knowledge of drug effects, and side effects), 12-Item Short Form Survey quality of life rating scale (SF-12), and Diabetes Treatment Satisfaction Questionnaire (DTSQ). Factors related to medication compliance were then evaluated. In all, there were 47 respondents: 31 males and 16 females. Four factors were found to be associated with medication compliance in elderly type 2 diabetic patients: medication storage (*P* = 0.01), knowledge of drug effects (*P* < 0.001), knowledge of side effects (*P* = 0.026), and physical functioning: (PF) (*P* = 0.045), a subscale of SF-12. Furthermore, the strength of the association between these four factors and medication compliance was calculated using Cramer's V coefficient of association. Knowledge of drug effects was the most strongly associated (knowledge of drug effects: V = 0.559; knowledge of side effects: V = 0.464; medication storage: V = 0.451; PF: V = 0.334). Because diabetes mellitus has no subjective symptoms and treatment effects are not felt to a great extent, it is difficult to motivate patients to consistently adhere to medication. When pharmacists provide medication guidance to elderly patients with type 2 diabetes mellitus, it is important to provide sufficient information to ensure they fully understand the drug effects to maintain medication compliance.

## Introduction

The increase in type 2 diabetes incidence is a serious problem worldwide (1). According to the International Diabetes Federation (IDF), the global number of patients with type 2 diabetes had reached 463 million by 2019 (2). About 4.2 million people die annually from diabetes complications (2, 3). The IDF has warned that if no effective measures are taken, the number of type 2 diabetes patients globally will reach 700 million by 2045 (2).

Many of the complications of diabetes, such as blindness, kidney disease, and neuropathy, are preventable (4). Nevertheless, patients continue to develop complications (5) because they do not experience any subjective symptoms (6), which makes it difficult to maintain motivation for long-term treatment. Therefore, many patients discontinue their medical visits (7) or have poor medication compliance (8).

Compliance is defined as the ability to consume the prescribed drug correctly as directed by the doctor and having a proper understanding of when to take it (9). Observing medication compliance is an important public health theme because it leads to prevention of aggravation of patients' condition, prevention of side effects such as hypoglycemia, and reduction of medical expenses (10).

In Japan, the number of patients with type 2 diabetes mellitus has increased rapidly due to changes in lifestyle and social environment. The incidence of associated complications has also increased markedly (5). The increasing need for dialysis in patients due to poor glycemic control results in a significant decrease in their quality of life (QOL) and increased pressure on national health care costs (11). Therefore, reducing the severity of diabetes is an important policy issue to reduce the national health care costs (12).

The focus of prevention of severe disease in patients with type 2 diabetes is to keep patients motivated to receive treatment (12). In many hospitals, each specialist provides support for hospitalized patients, such as diabetes treatment guidance by doctors, lifestyle guidance by nurses, drug treatment guidance by pharmacists, and nutrition guidance by dieticians (13, 14). However, most type 2 diabetes patients in Japan use clinics near their homes and receive support only from doctors. Accordingly, community pharmacists who receive out-of-hospital prescriptions play an important role in supporting drug treatment. In particular, local pharmacy pharmacists who receive out-of-hospital prescriptions play an important role, such as listening to the patient's compliance status and checking for the occurrence of side effects, through medication guidance. Regarding this, Article 1 of the Pharmacists Act stipulates that “A pharmacist is to contribute to the improvement and promotion of public health by administering the dispensing of medicine, supply of medicine and other pharmaceutical health and sanitation services, thereby ensuring the healthy living of citizens” (15). Therefore, the pharmacist's mission is to contribute to public health.

The average age of patients with type 2 diabetes in Japan is over 70 years old, reflecting the aging society (3). Pharmacists must be aware of the importance of providing guidance on drug therapy that considers the decline in patients' cognitive function and QOL associated with aging. Their support should aim to improve patients' medication compliance (16). Previous studies of patients with type 2 diabetes have shown that age and sex are risk factors for poor medication compliance (8, 17). Possible reasons why age is related to compliance include a decrease in understanding of drug efficacy and side effects (18) and a decrease in QOL as the elderly age (19). However, there are no reports that limit the survey population to the elderly, analyze factors related to medication compliance, and compare the strength of their effects. Therefore, it is necessary to clarify the strength of these factors to enable pharmacists to provide appropriate drug treatment support to aging patients in Japan. The prevention of severe disease in diabetic patients is also an important health-related global public policy issue (20).

This study investigated factors related to medication compliance in type 2 diabetes patients aged 65 years or older who visited pharmacies. A questionnaire survey was conducted on the following factors related to medication compliance: knowledge of the effects and side effects of diabetic drugs, patients' QOL status, and satisfaction with treatment. The purpose was to provide useful information for pharmacists to note when instructing elderly patients with type 2 diabetes by statistically clarifying the factors' strength related to medication compliance.

## Methods

### Study Design

This cross-sectional study was conducted using a self-completion questionnaire in patients who visited a dispensing pharmacy. The questionnaire was designed to take about 10 min to complete in consideration of the burden on the respondents.

### Participants and Settings

The study population was patients with type 2 diabetes aged 65 years or older who had not developed diabetic complications, or symptoms of complications, who self-managed their oral medications, did not have cognitive problems, and were able to complete the questionnaire without assistance. Data were collected from patients who visited dispensing pharmacies in the Shinagawa area (Tomato Pharmacy Futaba Store, Salad Pharmacy Togoshiginza Store, and Tanabe Pharmacy Shinagawa Ebara Store) to fill prescriptions at least once a month between March 1, 2019, and September 30, 2019. In this study, the sample size was not set in advance considering that the purpose was to evaluate the relative effect size of the factors related to compliance. The effect size was measured for the target population as elderly people who provided written informed consent within the study period. An explanation sheet describing the purpose of the survey was distributed and subjects who gave their consent were enrolled. Participants completed the questionnaire anonymously, answering questions related to patient demographics and medication compliance. Patients who could not fill out the questionnaire by themselves due to blindness, dementia, or other reasons were excluded from the study.

### Survey Items

The following information was collected: patient demographics, medication compliance status, knowledge of drug effects and side effects, health-related QOL, and treatment satisfaction.

Patient demographic information included age, sex, duration of diabetes, and number of medications taken.

**Figure 1:**
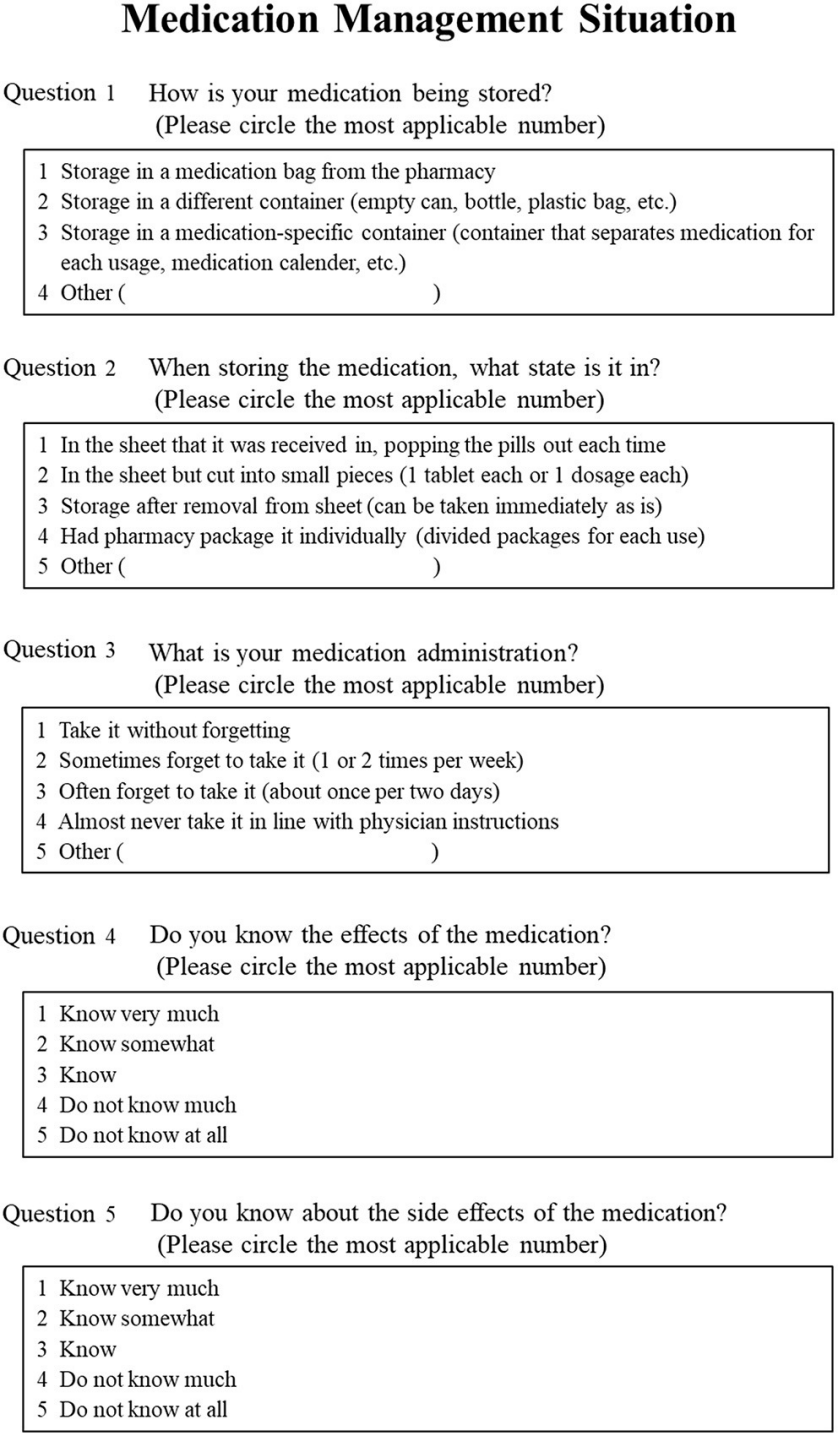
Survey regarding medication management situation.

To investigate medication compliance, the questionnaire in Figure 1 was used. We referred to the compliance questionnaire conducted on dialysis patients by Nagasawa et al. (21). The questionnaire used to investigate compliance in our study was considered relevant because dialysis patients are known to show inadequate compliance (22), and ~40% cases of dialysis in Japan can be attributed to nephropathy of type 2 diabetes (23). For the medication compliance status, “medication storage,” “medication management,” and “whether or not the patient forgets to take the medication” were surveyed. In this study, medication compliance (whether the patient takes the prescribed medication) was assessed instead of medication adherence. In terms of knowledge of drug effects and side effects, “understanding of drug effects” and “understanding of drug side effects” were surveyed.

For the measurement of QOL, we used SF-12v2, a widely used health-related QOL scale (24). It has eight subscales (Table 1). Each subscale was scored using the norm-based scoring (NBS) validated based on the national norms for SF-12v2. In addition, based on the eight subscales, three summary scores were calculated: “Physical component summary: PCS,” “Mental component summary: MCS,” and “Role/Social component summary: RCS.” Similarly, the summary scores were calculated by NBS scores. The NBS score was scored against the population standard based on a mean of 50 and a standard deviation of 10.

**Table 1:** Stratification criteria.

**Characteristics**		
Sex	Male	Female
Type of medications	≧5	<5
Frequency	≧2	<2
DTSQ score	≧Median	< Median
**SF-12**		
Subscale Score
PF	≧50	<50
RP	≧50	<50
BP	≧50	<50
GH	≧50	<50
VT	≧50	<50
SF	≧50	<50
RE	≧50	<50
MH	≧50	<50
Summary scores
PCS	≧50	<50
MCS	≧50	<50
RCS	≧50	<50

*PF, Physical functioning; RP, Role physical; BP, Bodily pain; GH, General health; VT, Vitality; SF, Social functioning; RE, Role emotional; MH, Mental health; PCS, Physical component summary; MCS, Mental component summary; RCS, Role/Social component summary*.

The Diabetes Treatment Satisfaction Questionnaire (DTSQ) was used to investigate satisfaction with diabetes treatment (25). The DTSQ is translated into more than 100 languages and is widely used in many countries because it is relatively easy to answer and can be used by treated and untreated patients.

### Stratification

**Figure 2:**
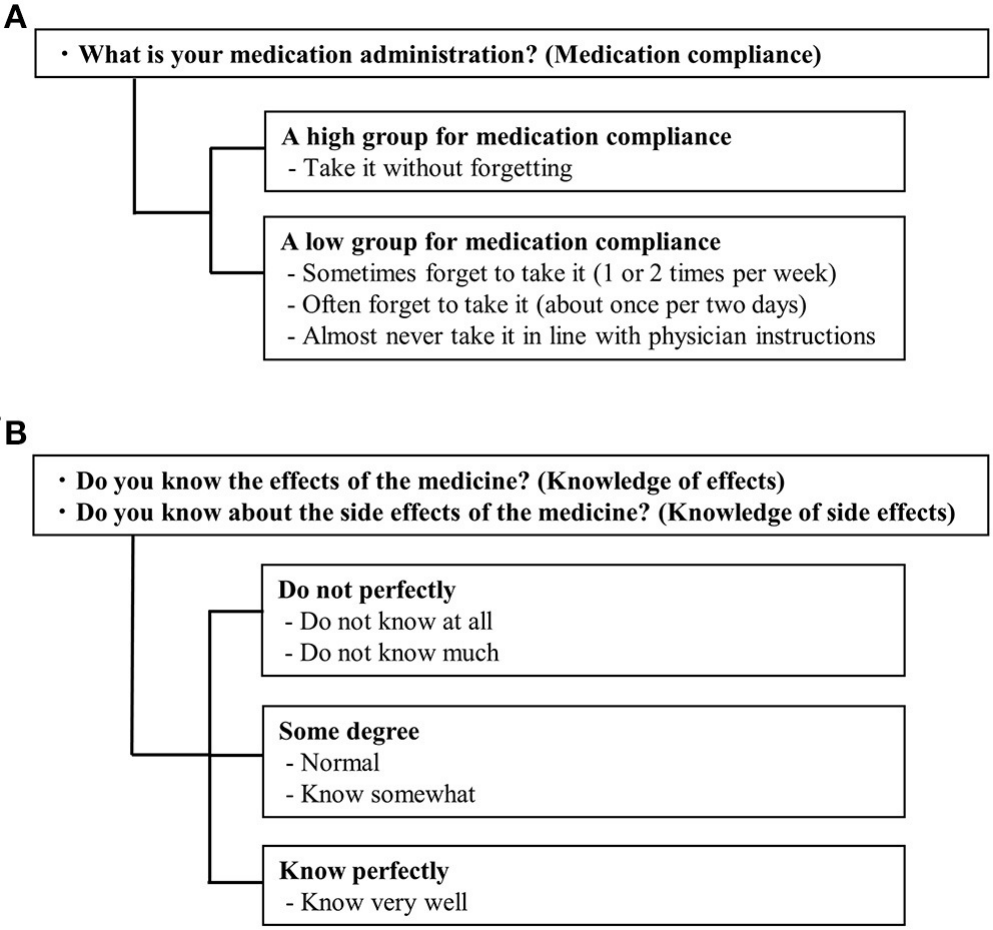
Stratification criteria for medication compliance and medication management situation. **(A)** Medication compliance. **(B)** Knowledge of effects and side effects.

The participants were grouped based on Figure 2 and Table 1, as a pre-treatment for the statistical analysis of the effect of each variable on medication compliance. Participants were stratified into high and low medication compliance groups based on their medication management status (Figure 2A). Patients who answered, “I never forget to take my medication,” were classified into the high medication compliance group, while those who responded with any of the other options were classified into the low medication compliance group. The patients were stratified for knowledge of drug effects and side effects according to the criteria in Figure 2B. Patient demographics, number of medications taken, frequency of taking medications, DTSQ score, and the eight subscales and three summary scores of SF-12 were also stratified according to Table 1.

### Statistical Analysis

Analyses were performed using pairwise elimination when any of the two variables had missing values to use as much data as possible. Differences in characteristics between the good and poor medication compliance groups were assessed by Fisher's exact test. The factors used were patient demographics, knowledge of drug effects and side effects, QOL, and treatment satisfaction (DTSQ). For items with a significant difference, Cramer's V coefficient of association was calculated to evaluate the strength of the association with medication compliance. The statistical software R version 4.0.3 (R Core Team, R Foundation for Statistical Computing, Vienna, Austria) was used. The significance level for all tests was set at *P* < 0.05.

### Ethical Considerations

This study was conducted with the approval of the Ethics Committee of Hoshi University (Approval No.: 30-022). The study was conducted in accordance with the Declaration of Helsinki Ethical Guidelines for Medical Research Involving Human Subjects. Written informed consent was obtained from all participants.

## Results

### Patient Information

The patient information is shown in Table 2. There were 47 participants. Of these, three patients did not respond to the duration of disease item, and 4 patients did not respond to the DTSQ item. The mean age of the participants was 75.0 ± 5.7 years old, and 31 (66.0%) were male. The median duration of disease was 8.0 years (interquartile range: 2.9–20.0), and the median DTSQ was 29.0 (interquartile range: 25.0–32.5).

**Table 2:** Patient characteristic.

	***n* (%)**
**Age (*****n*** **=** **47)**
Average ± standard deviation	75.0 ± 5.7
**Sex (*****n*** **=** **47)**
Male	31 (66.0)
Female	16 (34.0)
**Disease duration (*****n*** **=** **44)**
Median value	8.0
(interquartile range)	(2.9–20.0)
**DTSQ score (*****n*** **=** **43)**
Median value	29.0
(interquartile range)	(25.0–32.5)

*DTSQ, Diabetes Treatment Satisfaction Questionnaire*.

### Medication Management Status

The results of the medication management status are listed in Table 3. The number of non-responses was: six for type of medication, three for storage, three for compliance, three for knowledge of drug effects, and three for side effects.

**Table 3:** Survey results (medication management situation).

**Medication management situation**	***n* (%)**
**Type of medications (*****n*** **=** **41)**	
1–3 types medicine	10 (24.3)
4–6 types medicine	17 (41.5)
7–9 types medicine	4 (9.8)
10 types or more	11 (26.8)
**Frequency (*****n*** **=** **47)**	
Median value	2.0
Interquartile range	(2.0–3.0)
**Medication storage (*****n*** **=** **44)**	
Medication bag	10 (22.7)
Different container (empty can, bottle, plastic bag, etc.)	25 (56.8)
Medication-specific container (container separating medication by usage, medication calendar, etc.)	7 (15.9)
Other	2 (4.5)
**Medication storage state (*****n*** **=** **42)**	
In the sheet as it was received, poping the pill out each time	14 (33.3)
In the sheet but cut into small pieces (1 tablet each or 1 dosage each)	13 (31.0)
Removed from sheet (unwrapped pills)	9 (21.4)
Packaged individually by pharmacy	6 (14.3)
**Compliance (*****n*** **=** **44)**	
Take it without forgetting	34 (77.3)
Sometimes forget to take it (1 or 2 times per week)	3 (6.8)
Often forget to take it (about once per 2 days)	6 (13.6)
Almost never take it in line with physician instructions	0 (0)
Other	1 (2.3)
**Knowledge of effects (*****n*** **=** **44)**	
Know very much	14 (31.8)
Know somewhat	20 (45.5)
Know	7 (15.9)
Do not know much	2 (4.5)
Do not know at all	1 (2.3)
**Knowledge of side effects (*****n*** **=** **44)**	
Know very much	7 (15.9)
Know somewhat	16 (36.4)
Know	7 (15.9)
Do not know much	11 (25.0)
Do not know at all	3 (6.8)

In the question on types of medication, 17 patients (41.5%) answered “4–6 types,” and the average frequency of taking medication was 2.0 ± 1.2 times.

Regarding the medication storage method, most of the respondents, 25 (56.8%), answered “Store them in different containers (empty cans, bottles, and plastic bags),” and regarding the storage condition of the medication, the largest number of respondents, 14 (33.3%), answered, “Store them in sheets.”

Regarding medication compliance, 34 respondents (77.3%) answered that they take their medication without forgetting. Regarding knowledge of drug effects and side effects, the largest number of respondents, 20 (45.5%) for knowledge of drug effects and 16 (35.4%) for knowledge of side effects answered “Have knowledge about both items to some extent.”

### Assessment of QOL

**Figure 3:**
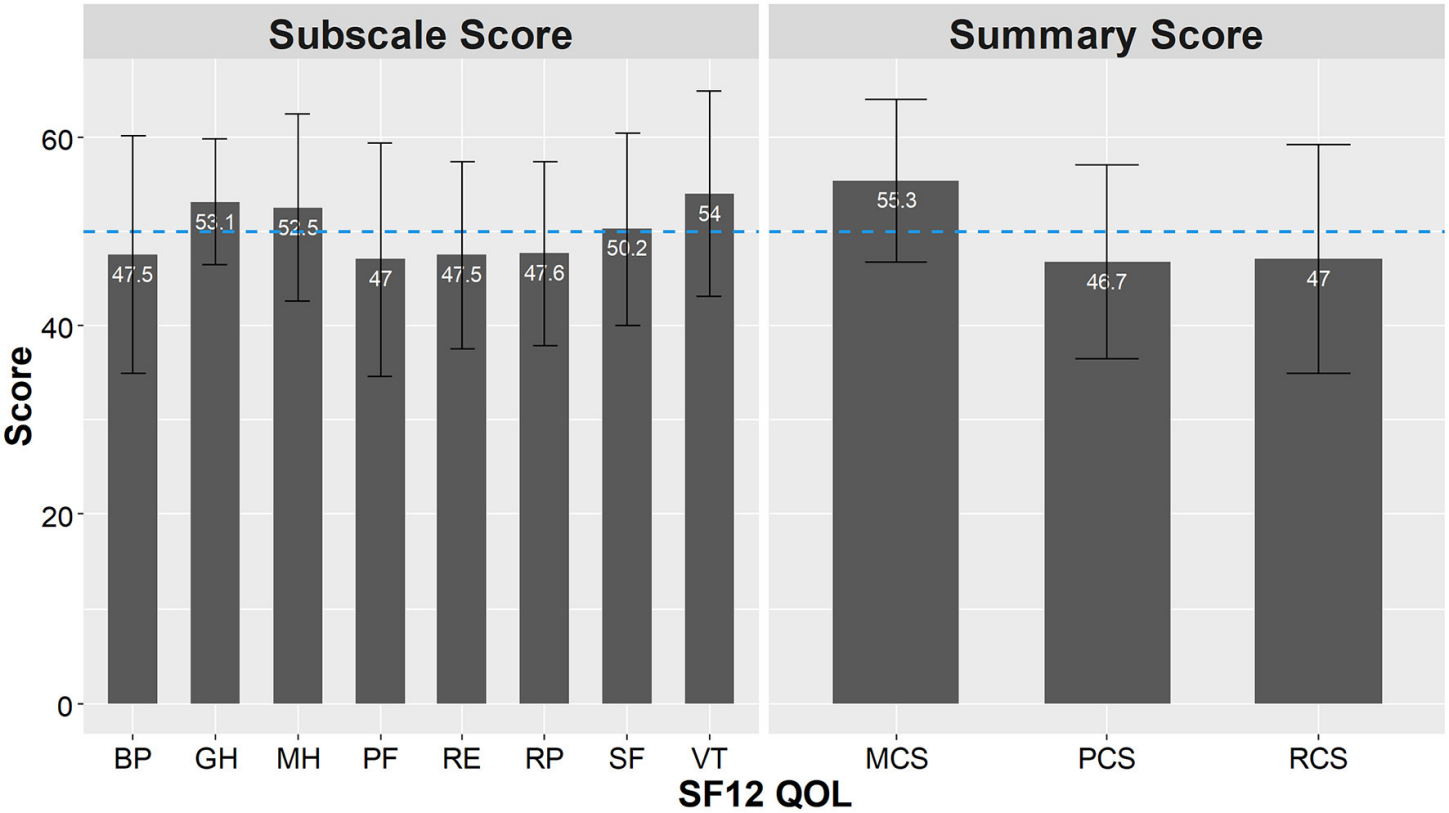
Analysis of SF-12 subscales scores and summary scores. SF-12 subscales scores and summary scores based on the norm-based scoring (NBS). PF, physical function; RP, role physical; BP, bodily pain; GH, general health; VT, vitality; SF, social function; RE, role emotional; MH, mental health; PCS, physical component summary; MCS, mental component summary; RCS, role component summary.

The results of the SF-12 are shown in Figure 3. Among the eight subscales, Physical Functioning (PF) 47.0 ± 12.3, Role Physical (RP) 47.6 ± 9.7, Bodily Pain (BP) 47.5 ± 12.6, and Role Emotional (RE) 47.5 ± 9.9, were below the national standard. General Health (GH) 53.1 ± 6.7, Vitality (VT) 54.0 ± 10.9, Social functioning (SF) 50.2 ± 10.2, and Mental Health (MH) 52.5 ± 9.9, were above the national standard. Concerning the summary scores, Mental Component Summary (MCS) 55.3 ± 8.6, was higher than the Japanese standard, and Physical Component Summary (PCS) 46.7 ± 10.3, and Role/Social Component Summary (RCS) 47.5 ± 9.9, were lower than the Japanese standard.

### Evaluation of Factors Related to Medication Compliance

Regarding the association between patient characteristics and QOL in the groups with good and poor medication compliance, the results of the assessment using Fisher's exact test are shown in Table 4.

**Table 4:** Analysis of related factors for medication compliance.

	**Low group**	**High group**	***P***
	***n* (%)**	***n* (%)**	
**Sex**			1.00
Men	9 (19.1)	22 (46.8)	
Women	4 (8.5)	12 (25.5)	
**Duration**			0.32
≥ Median	8 (18.2)	15 (34.1)	
< Median	4 (9.1)	17 (38.6)	
**Frequency**			1.00
≥ Median	11 (23.4)	27 (57.4)	
< Median	2 (4.3)	7 (14.9)	
**Type of medication**			0.37
1–3 types medicine	2 (4.8)	8 (19.0)	
4–6 types medicine	8 (19.0)	9 (31.9)	
7–9 types medicine	1 (2.4)	3 (7.1)	
10 types medicine	2 (4.8)	9 (21.4)	
**Medication storage**			**0.01**
Medication bag	3 (6.8)	7 (15.9)	
Different container (empty can, bottle, plastic bag, etc.)	2 (4.5)	23 (52.3)	
Medication-specific container (container separating medication by usage, medication calendar, etc.)	4 (9.1)	3 (6.8)	
Other	1 (2.3)	1 (2.3)	
**Medication storage state**			0.64
In the sheet as it was received, popping the pill out each time	2 (4.8)	12 (28.6)	
In the sheet but cut into small pieces (1 tablet each or 1 dosage each)	3 (7.1)	10 (23.8)	
Removed from sheet (unwrapped pills)	3 (7.1)	6 (14.3)	
Packaged individually by pharmacy	2 (4.8)	4 (9.5)	
**Knowledge of effects**			***P*** **<** **0.001**
Know perfectly	0 (0.0)	14 (29.8)	
Some degree	8 (17.0)	19 (40.4)	
Not understanding	5 (10.6)	1 (2.1)	
**Knowledge of side effects**			**0.026**
Know perfectly	1 (2.1)	6 (12.8)	
Some degree	4 (8.5)	19 (40.4)	
Not understanding	5 (10.6)	9 (19.1)	
**SF-12**			
Subscale score			
PF			**0.045**
≥50	4 (8.5)	23 (48.9)	
<50	9 (19.1)	11 (23.4)	
RP			0.19
≥50	4 (8.5)	19 (40.4)	
<50	9 (19.1)	15 (31.9)	
BP			0.11
≥50	4 (8.5)	20 (42.6)	
<50	9 (19.1)	14 (29.8)	
GH			0.48
≥50	12 (25.5)	33 (70.2)	
<50	1 (2.1)	1 (2.1)	
VT			0.70
≥50	11 (23.4)	26 (55.3)	
<50	2 (4.3)	8 (17.0)	
SF			0.52
≥50	7 (14.9)	22 (46.8)	
<50	6 (12.8)	12 (25.5)	
RE			0.34
≥50	5 (10.6)	19 (40.4)	
<50	8 (17.0)	15 (31.9)	
MH			0.75
≥50	8 (17.0)	18 (38.3)	
<50	5 (10.6)	16 (34.0)	
Summary scores			
PCS			0.10
≥50	3 (6.4)	18 (38.8)	
<50	10 (21.3)	16 (34.0)	
MCS (≥ 50)			0.30
≥50	11 (23.4)	23 (48.9)	
<50	2 (4.3)	11 (23.4)	
RCS (≥ 50)			0.52
≥50	6 (12.8)	20 (42.6)	
<50	7 (14.9)	14 (29.8)	
**DTSQ**			0.52
≥ Median	6 (12.8)	20 (42.6)	
< Median	7 (14.9)	14 (29.8)	

Sex, duration of diabetes mellitus, type of medication taken, medication management status, DTSQ, and three summary scores of SF-12 were not significantly associated with medication compliance.

On the other hand, medication storage status (*P* = 0.01), PF (a subscale of SF-12) (*P* = 0.045), knowledge of drug effects (*P* < 0.001), and knowledge of side effects (*P* = 0.026) were associated with medication compliance.

### Assessment of the Strength of Factors Related to Medication Compliance

Cramer's V coefficients of association were calculated to determine the degree of association of factors with medication compliance. Knowledge of drug effects (V = 0.559), knowledge of side effects (V = 0.464), medication storage (V = 0.451), and the SF-12 subscale PF (V = 0.334) were strongly associated with medication compliance, in that order (Table 5).

**Table 5:** Strength of relationship with medication compliance.

	**Cramer's V**
Knowledge of effects	0.559
Knowledge of side effects	0.464
Medication storage	0.451
PF	0.334

## Discussion

Our results showed four factors were related to medication compliance in elderly type 2 diabetic patients: medication storage status, knowledge of drug effects, knowledge of side effects, and the SF-12 subscale PF. Among these factors, knowledge of drug effects showed the strongest association with medication compliance. However, sex, subscales of SF-12 other than PF, summary score, the DTSQ rating scale, and medication management status were not associated with medication compliance.

The association between sex and compliance is unclear. There are reports that compliance is negatively correlated with sex, especially in men (26–28) and also that it has no association with sex (29–35). Our results showed no association. The patient characteristics of this study, 66% male and an average age of 75.0, is almost the same as the characteristics of diabetes patients in Japan. From this, it is inferred that the sampling of diabetic patients was less biased. In addition, the results may differ depending on the survey conditions and environment.

Concerning the SF-12, previous results of studies on dialysis patients (36) and hypertensive patients (37) have shown that its subscale, PF, is significantly associated with medication compliance, and the present study also found an association. This means that regardless of the type of disease, PF may be associated with medication compliance. Therefore, it is important to be aware that patients with decreased PF may have decreased compliance, when providing medication guidance.

No significant difference was found in the association between DTSQ and compliance. A previous study examined whether treatment satisfaction affected compliance for patients taking DPP-4 anti-diabetes drugs. Although there was an association between lower blood glucose levels and treatment satisfaction, there was no association with compliance (38). Although this study did not consider the specific drug type or management method of the diabetes medication, our results are similar in finding no relationship between treatment satisfaction and compliance. Therefore, health care providers should not assume that because patients are satisfied with their treatment, they are taking their medication as prescribed.

There was a tendency toward better compliance in patients who stored their medication in a separate container rather than the bag they received from the pharmacy. It can be assumed that people who have a strong awareness of managing their medication are more active in their treatment, which is related to good medication compliance. On the other hand, the method of medication management was not significantly associated with compliance. Patients' compliance with medication can be improved if medical professionals such as pharmacists take measures, such as packaging all of a patient's drugs (39, 40). However, no matter how much effort is made by medical professionals to help patients on chronic medication, if the patients do not understand the significance of taking the medications continuously, their compliance will decrease in the long run (41). The longer patients are on medication, the more likely they will stop taking their medication without medical guidance. Therefore, health care providers should not rely only on measures such as packaging but should appropriately evaluate and respond to the patient's situation.

The association between knowledge of drug effects and medication compliance results from patients not feeling the drug effects. Owing to the fact that diabetes is a chronic disease that progresses even in the absence of subjective symptoms (42), patients do not feel effects of the drug even if they consume it continuously unless the patient measures blood glucose. As a result, they stop taking it because of the daily inconvenience. Therefore, it is important to thoroughly explain to patients how long they should take the medication, how much their blood glucose levels will improve, and what the benefits are.

In our study, in the group with good medication compliance, the percentage of patients who knew both the drug effects and side effects “well” or “to some extent” was high. Conversely, in the group with poor medication compliance, the percentage of patients who did “not know” was high. Further, knowledge of drug effects was more strongly associated with medication compliance than knowledge of side effects (Cramer's V: knowledge of drug effects: 0.559 > knowledge of side effects: 0.464). This interesting finding suggests that it is important to properly instruct patients on the effects of the drugs they have been prescribed to maintain good medication compliance. For anticancer drugs, which have a very high incidence of serious side effects, knowledge of side effects is important in maintaining patients' medication compliance (43). By contrast, drugs for chronic diseases, such as diabetes medications, have a low risk of serious side effects. However, improvement in patients' knowledge of drug effects influences their adherence (44, 45). Therefore, it is especially important for elderly patients with type 2 diabetes to properly understand the effects of their drugs to maintain good medication compliance.

This study has several limitations. The first is that the study population was small (47 patients). Since the inclusion criteria included elderly diabetic patients without complications or other underlying diseases, it was difficult to ensure enough cases. As a result, there may be a decrease in the power of the test, i.e., Type 2 errors may have increased. Second, this was a cross-sectional study, therefore, it could not prove a causal relationship. The third is that there were many missing values in the data. This may be due to the advanced age of the respondents. Pairwise deletion was used in the analysis to minimize the reduction in the number of cases. This may have introduced some bias. Fourth, in the present study, we conducted a survey limited to what Japanese pharmacists actually check when giving medication guidance; therefore, we did not ask questions about the patient's socioeconomic condition, education level, diet plan, and so on. Fifth, our survey was conducted with individuals who could provide informed consent in writing. Therefore, the compliance status of patients who did not wish to provide informed consent may not have been reflected at accurately. However, despite the small number of participants, this study was able to show that medication knowledge was more strongly associated with medication compliance than other factors. In particular, it was shown that knowledge of the effects of drugs is more important than providing knowledge of side effects or devising dispensing methods. This is a very interesting finding when medical professionals such as pharmacists instruct patients. It is important to emphasize this to physicians and pharmacists who wish to maintain good medication compliance in type 2 diabetic patients.

Given the increasing number of diabetic patients, including those with complications, pharmacists must fully inform patients about drug efficacy and help them understand the drug effects to contribute to improving their medication compliance.

## Conclusion

Knowledge of drug effects is important to improve medication compliance in elderly type 2 diabetic patients. When providing patients with information about medications, it is important to provide specific explanations about the drug effects more than its side effects to maintain good medication compliance.

## Data Availability Statement

The original contributions presented in the study are included in the article/supplementary material, further inquiries can be directed to the corresponding author/s.

## Ethics Statement

The studies involving human participants were reviewed and approved by Ethics Committee of Hoshi University. The patients/participants provided their written informed consent to participate in this study.

## Author Contributions

MO, TY, and MK collected data. NW and SE analyzed the data. NW wrote the manuscript. SS and YM critically revised it for important intellectual content. All authors contributed to the study design, read, and approved the manuscript.

## Conflict of Interest

The authors declare that the research was conducted in the absence of any commercial or financial relationships that could be construed as a potential conflict of interest.

## Publisher's Note

All claims expressed in this article are solely those of the authors and do not necessarily represent those of their affiliated organizations, or those of the publisher, the editors and the reviewers. Any product that may be evaluated in this article, or claim that may be made by its manufacturer, is not guaranteed or endorsed by the publisher.
